# Light-Weight Deep Learning Techniques with Advanced Processing for Real-Time Hand Gesture Recognition

**DOI:** 10.3390/s23010002

**Published:** 2022-12-20

**Authors:** Mohamed S. Abdallah, Gerges H. Samaan, Abanoub R. Wadie, Fazliddin Makhmudov, Young-Im Cho

**Affiliations:** 1 Department of Computer Engineering, Gachon University, Seongnam 1342, Republic of Korea; 2 Informatics Department, Electronics Research Institute (ERI), Cairo 11843, Egypt; 3 Department of Computer Science, Faculty of Computers and Artificial Intelligence, Helwan University, Helwan 11731, Egypt

**Keywords:** hand gesture, sign language, MediaPipe, hand landmarks, GRU, 1D CNN, DSL-46

## Abstract

In the discipline of hand gesture and dynamic sign language recognition, deep learning approaches with high computational complexity and a wide range of parameters have been an extremely remarkable success. However, the implementation of sign language recognition applications for mobile phones with restricted storage and computing capacities is usually greatly constrained by those limited resources. In light of this situation, we suggest lightweight deep neural networks with advanced processing for real-time dynamic sign language recognition (DSLR). This paper presents a DSLR application to minimize the gap between hearing-impaired communities and regular society. The DSLR application was developed using two robust deep learning models, the GRU and the 1D CNN, combined with the MediaPipe framework. In this paper, the authors implement advanced processes to solve most of the DSLR problems, especially in real-time detection, e.g., differences in depth and location. The solution method consists of three main parts. First, the input dataset is preprocessed with our algorithm to standardize the number of frames. Then, the MediaPipe framework extracts hands and poses landmarks (features) to detect and locate them. Finally, the features of the models are passed after processing the unification of the depth and location of the body to recognize the DSL accurately. To accomplish this, the authors built a new American video-based sign dataset and named it DSL-46. DSL-46 contains 46 daily used signs that were presented with all the needed details and properties for recording the new dataset. The results of the experiments show that the presented solution method can recognize dynamic signs extremely fast and accurately, even in real-time detection. The DSLR reaches an accuracy of 98.8%, 99.84%, and 88.40% on the DSL-46, LSA64, and LIBRAS-BSL datasets, respectively.

## 1. Introduction

Nowadays, over 5% of the world’s population are hearing-impaired. This percentage is expected to be 10% by 2050 [1,2]. The hearing impaired community is expected to reach 2.5 billion by 2050, and one in every ten people will be affected by disabling hearing loss. Those people suffer every day in communication with society.

The hearing impaired communities often use sign language, which is a system that uses visual-manual modality to convey a meaning. Sign language depends mainly on hand gestures, body movements, and facial expressions. Sign language recognition (SLR) is a challenging task, especially the recognition of dynamic signs that depends on movement. This is why many researchers are interested in developing an SLR application with the goal of decreasing the barrier between hearing impaired communities and society.

Sign language recognition methodologies are usually divided into two categories: static and dynamic [3,4,5]. Static signs are those that only require the processing of a single image at the input of the classifier; hence, it can be treated as a screenshot of the hand shape. Dynamic signs can be treated as a video containing a number of consecutive frames to construct a sign. Generally, in sign language, the signs are built from a series of quick hand actions and body expressions; hence, static sign language recognition is not a good solution for sign language problems as it can not deal with the variation of signs. Therefore, the dynamic-based solution is more effective and efficient for solving sign language problems.

There are some challenges that face the SLR that can be classified as primary and secondary factors [6,7,8].

1. The primary factors:
  - Hand Shape: The difference in the shape of the hand changes the sign;
  - Hand Location: The hand location relative to the body can change the meaning of the sign even if the hand shape is the same;
  - Hand Movement: The most complex parameter as the sign can contain a set of movements with different directions and shapes.
2. The secondary factors:
  - Facial Expressions: The expressions on the face of the signer play a vital role in illustrating the sign; it increases the sense and strength of the meaning in the communication process.
  - Orientation of the palm: The direction of the palm when making a sign—whether it is facing up or down, right, or left.

Although the secondary factors are not essential in recognizing the sign, it is preferred to take them into consideration. All the primary and secondary factors work together to give the mean of a sign; they are complementary.

Our paper presents a solution that can deal with both primary and secondary factors to accurately recognize the sign. We propose a Dynamic Sign Language Recognition approach by combining deep learning models with the MediaPipe framework.

Our approach provides two reliable deep learning models for Dynamic Sign Language Recognition (DSLR). The first one is the Recurrent Neural Network (RNN) model: Gated Recurrent Unit (GRU), while the second is the 1DCNN model [9,10,11].

The MediaPipe was utilized to offer details on the palm’s orientation, hand location, hand shape, and track them [12,13]. In addition, it is possible to accurately recognize signs by extracting facial expressions using the face mesh approach (Appendix A).

Although MediaPipe succeeded in extracting all the needed information from the primary and secondary factors, DSLR is still a challenging task due to the difficulties facing computer vision, which particularly impairs the real-time detection of sign language. Some of those difficulties are:

- The change in the location of the signer: e.g., if the model is trained where the position of a signer in a specific sign is always to the right, it may fail to recognize this sign if the signer stands to the left in real-time;
- Depth of the body in the frame: it is the distance of the body from the camera, which also affects recognition negatively if changed from what the model was trained on;
- The unbalanced number of frames: the number of frames that will be used as input for the model should be standardized;
- Provide a dataset for training.

To solve these problems, we propose new algorithms to deal with the problems related to real-time detection, e.g., location and depth processing. We conduct 12 experiments on the GRU model and 1D CNN model with pose keypoints included or excluded.

In addition, we recorded a new dataset of dynamic sign language that contains 46 daily used vocabularies (signs) named DSL-46. We also used external datasets, which are LSA64 [14] and LIBRAS-BSL [8,15], so that we could compare our results with other recent research that used the same datasets.

Finally, the proposed solution could achieve excellent accuracy in recognizing dynamic signs, as shown in the experiments.

The following succinctly summarizes the main characteristics that distinguish our work approach from the other ways in the literature:

- The work approach stands out in its being light-weight and it performing faster than most of the state-of-the-art SLR methods in both training and testing with an accuracy of around 99%;
- The method works very well in real-time detection, which is our main goal of the research; it detects signs quickly and accurately;
- The method has a preprocessing algorithm that deals with the complexity between the actual input data and the test data, e.g., a frame selection algorithm, a scale algorithm, and a location processing algorithm; these algorithms allow the model to accurately determine the sign in the video even if the location and size of the signer or the length of the video were different from the training;
- The method does not need high-end computational power to work; it can work very smoothly on medium-end PCs or laptops;
- The method does not contain complex mathematics and calculations in comparison to others.

In the rest of this paper, we carry out the steps and the methodology of our solution method. Section 2 shows the related work. Section 3 proposes system details, while Section 4 explains the details of our dataset. In Section 5, the experiments are discussed. Finally, the conclusions in Section 6 are a summary of the entire research. In addition, Appendix A shows the face mesh method.

## 2. Related Work

The research articles for sign language recognition will be discussed in this section to compare them to the state-of-the-art, especially with those who used the same datasets.

Motion trajectory and hand shapes are two traditional approaches to DSL recognition problems. The studies [16,17,18,19] used motion trajectory data from sensors such as the electrical glove, gyroscope, and Kinect to categorize hand motions. These methods are limited to just a few basic hand motions, such as waving, raising, and lowering the hand.

In the past decade, several methods for dynamic hand gestures of sign language recognition have been proposed using deep learning algorithms such as RNNs [9,10,11,20], CNNs [21,22,23,24,25,26], and RCNNs [27,28].

Indian sign language (ISL) gesture recognition using CNN with selfie mode sign language video methodology was demonstrated by Rao et al. [26]. A recurrent convolutional neural network (RCNN) utilizing video for dynamic hand sign recognition was developed by Cui et al. [27].

The purpose of this section is to describe the research and methodologies that were used on the same datasets that we used for our experiments, which are the DSL-10, DSL-46, LSA64, and LIBRAS-BSL datasets. In Section 4 and Section 5.1, these datasets will be thoroughly explained.

Ronchetti et al. [29] proposes a broad probabilistic sign classification model that includes sub-classifiers based on several sorts of information such as position, movement, and hand shape. To test the hypothesis that ordering is not necessary for recognition, the model adopts a bag-of-words strategy in all classification steps. A separate sub-classifier is used for each hand, and the model integrates the results from both. The series of segmented hand images and hand locations is recovered from the sample video. The segmented hand pictures are utilized as input for the hand shape sub-classifier, while the position information for each hand is provided to the position and movement sub-classifiers. The final output includes the probabilities produced by the right-hand and left-hand sub-classifiers for each class.

Ramos et al. [30] recognize sign language using 3DCNN, standardize, and fix the number of frames using Nearest Neighborhood Interpolation (NNI) [31].

In addition, those who followed the approach of the CNN are Escobedo et al. [8] where they depended on describing the position and movement of the hand. Their method used texture maps to encode multimodal information (RGB-D). They presented a simple technique for obtaining a frame that accurately captures the shape of the hand. They then applied these data as inputs to two CNN models—one with three streams and the other with two streams—to learn robust features capable of identifying a dynamic sign. Two sign language datasets (LSA64 and LIBRAS-BSL) were used in the experiments and compared with state-of-the-art SLR methods.

Konstantinidis et al. [32] relied on VGG16 to extract hand and body skeleton data before applying a deep learning classifier to detect the sign.

Konstantinidis et al. [33] once more attempted to recognize sign language, but this time in stronger, more organized ways. They used three parallel extractions—first, image feature extraction using VGG16, then optical flow extraction using FlowNet2, and finally skeleton extraction using OpenPose—and then entered each one separately into its own LSTM model before concatenating them all to extract the result.

With the use of MediaPipe, which was used to estimate the location, shape, and orientation, Samaan et al. [20] developed three RNN models in order to address the issue of frame dependency in sign movement. Comparisons were made between the usage of face keypoints and their removal, as well as between the various RNN model types, such as GRU, LSTM, and BI-LSTM. The comparisons were conducted using the DSL-10 dataset, which is a subset of the larger DSL-46 dataset that was created during this study.

## 3. Methodology

**Figure 1:**
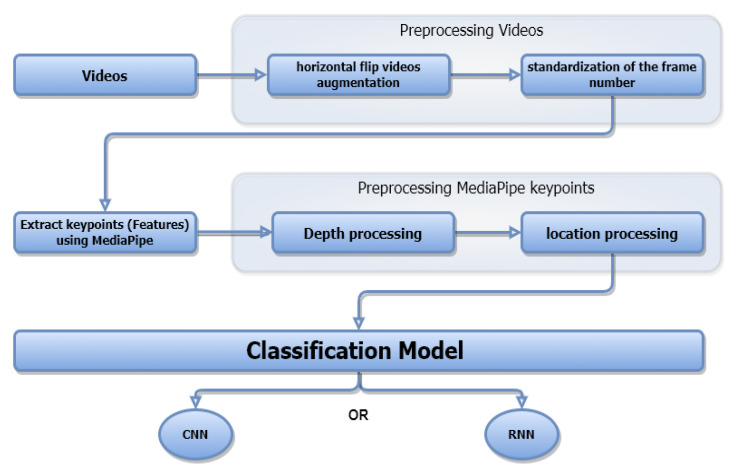
Overview of our proposed Dynamic Sign Language Recognition system.

When attempting to address the dynamic sign language recognition challenges, our approach goes through a series of three ordered phases until it obtains the optimal outcomes. The first phase begins with gathering and processing data such as video augmentation and standardization of frame number. The second phase is for extracting the keypoints using the MediaPipe framework, then processing the retrieved keypoints such as non-standard depth and location. In the last phase, we ultimately develop two deep learning models that can process consecutive input types. Figure 1 depicts the workflow for the full solution.

### 3.1. Collecting Data

We created a new dynamic sign language dataset called DSL-46, which includes 46 commonly used vocabulary. LSA64 and LIBRAS-BSL, two additional datasets, were also utilized to evaluate the performance and accuracy of our models.

### 3.2. Preprocessing Videos

#### 3.2.1. Augmentation

**Figure 2:**
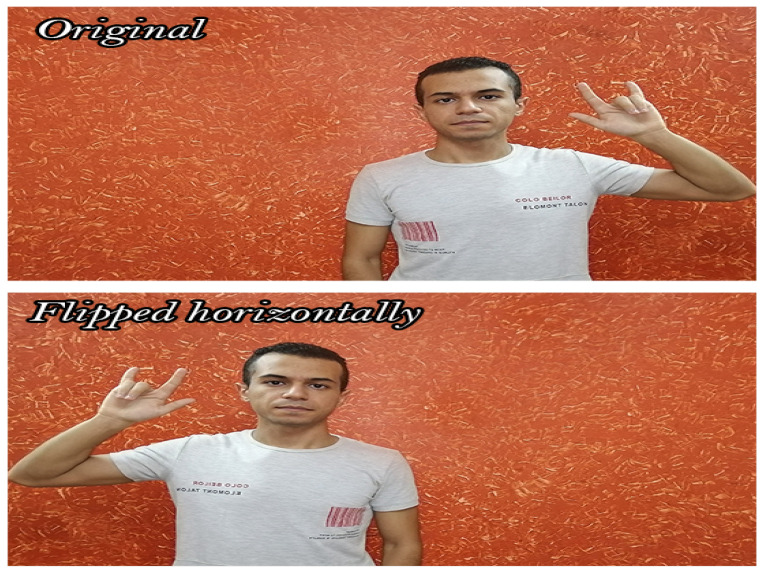
Flip horizontal augmentation.

Throughout this step, all videos in the DSL-46 dataset are horizontally flipped to accomplish two main objectives. First, broaden the data collected to improve testing and training procedures. Second, make sure that there are enough videos for both the left and right hands for the same sign. Figure 2 shows an example of flip horizontal augmentation.

#### 3.2.2. Standardize Frames Number (Solve Unbalanced Data)

**Figure 3:**
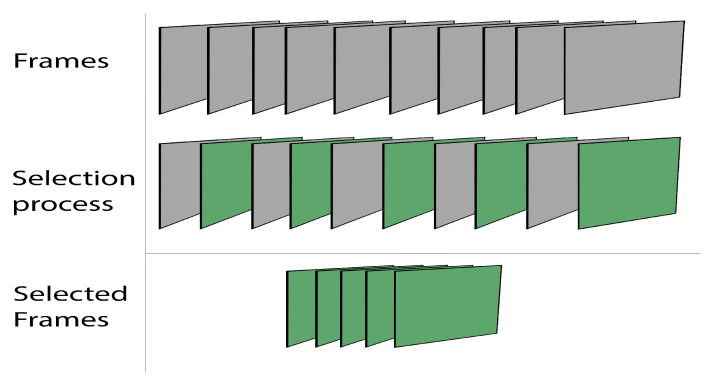
Selection process.

We equalize the number of frames in each video at this step in order to make the video suitable for use as input to the model, as illustrated in Figure 3.

The following Algorithm 1 determines whether the original video’s frame number is fewer or more than the desired number of frames. In that scenario, it is doubled or a certain number of frames are removed until the desired number is obtained.
**Algorithm 1 **Frame selection**Require:** 
the original video($v_{o}$) which is list of frames.**Require:** the number of frames to be extracted($F_{n}$).1:$v{}_{n}{}_{e}{}_{w}\leftarrow List{}_{E}{}_{m}{}_{p}{}_{t}{}_{y}$2:$count_{F}=Size\left(v_{o}\right)$3:I←14:**while **$I\neq (F_{n}+1)$** do**5:    $idx\leftarrow Integer(\left(i/F_{n}\right)*count_{F})$6:    $frame\leftarrow v_{o}.get\left(idx\right)$7:    $v{}_{n}{}_{e}{}_{w}.append\left(frame\right)$8:    I:=I+19:**end while**10:**return** video ${}_{f}{}_{i}{}_{x}{}_{e}{}_{d}$

### 3.3. Extract Keypoints Using MediaPipe

**Figure 4:**
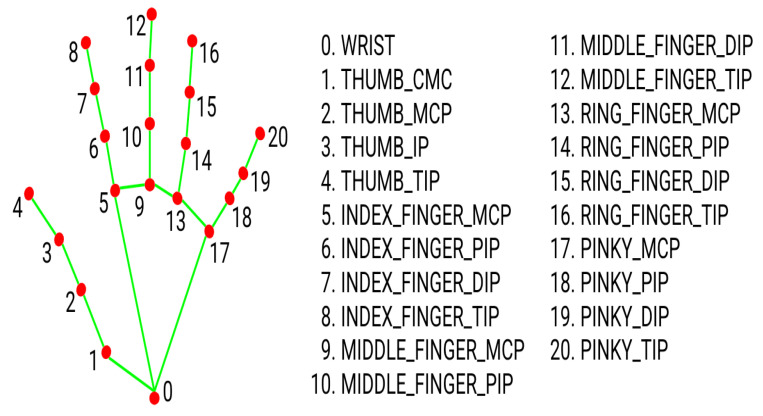
The order and labels of MediaPipe’s Hands keypoints [20].

In this step, the videos are now prepared to extract hands and pose keypoints (features) using MediaPipe. For both hands, the number of keypoints extracted from one frame is 126, since the MediaPipe extracts keypoints in three dimensions (*x*, *y*, and *z*-axes), with 21 keypoints for each dimension [12]. Figure 4 shows the 21 extracted hand’s keypoints. The extraction model is very well trained in that it can even obtain the coordinates of partially visible hands.

**Figure 5:**
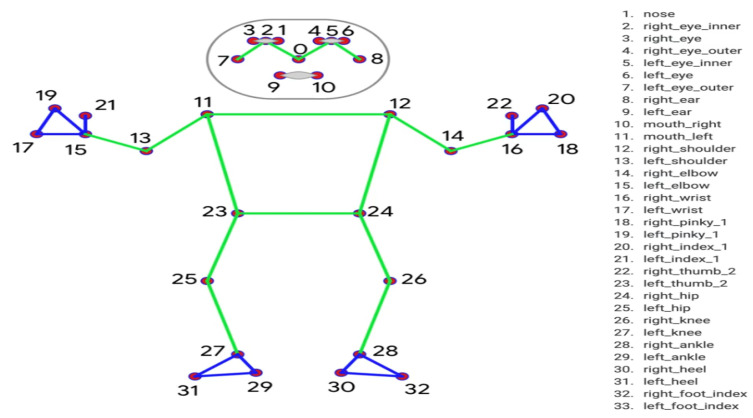
The order and labels of the pose’s keypoints [20].

For the pose, the number of keypoints extracted from one frame is 132, since the MediaPipe extracts 33 keypoints for each dimension plus the visibility [13]. The visibility of a point is a value that indicates whether it is visible or concealed (occluded by another body part). The 33 extracted pose keypoints are shown in Figure 5. The extracted keypoints for each video are saved in a CSV file. Now, the model can locate the hands by determining their shape, direction, and position relative to the body.

### 3.4. Preprocessing MediaPipe

#### 3.4.1. Scale (Depth Processing)

**Figure 6:**
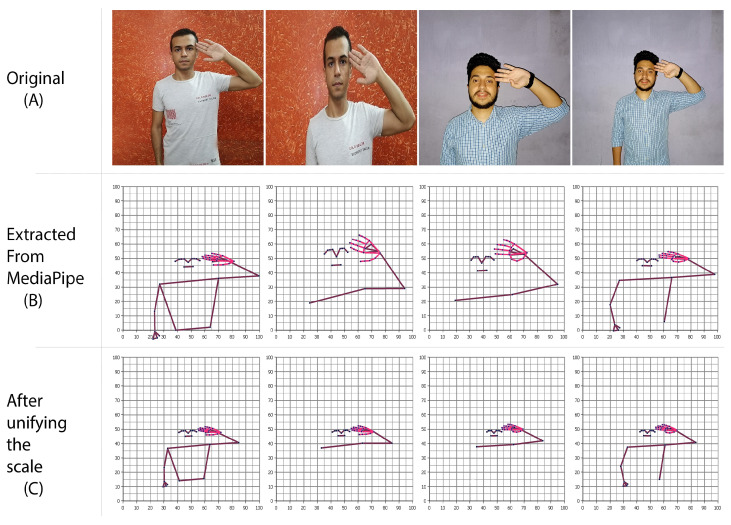
Depth processing.

Once the videos were used as input data for MediaPipe and the keypoints were extracted, the model encounters a new issue as a result of the variation in the depth of the object in the image (the object was zoomed in or out through the camera lens) as shown in Figure 6A.

The depth variation makes it difficult to recognize signs, especially in real-time because objects at the distance take up less space than those in the vicinity.

To solve this issue, first, identify the area of the object that was extracted using the MediaPipe and then unify a fixed area (depth) for all objects. Finally, either reduce or enlarge the portion of the object that was extracted to match the same area that was identified for all objects. The following Algorithm 2 shows the logical steps to solve this issue.
**Algorithm 2 **Scale Algorithm**Require:** 
Get all X and Y axes points Extracted from MediaPipe.**Require:** 
Get Highest and lowest points for both X and Y.**Require:** 
Define constant number for the width and height length.1:$width\leftarrow x{}_{m}{}_{a}{}_{x}-x{}_{m}{}_{i}{}_{n}$2:$height\leftarrow y{}_{m}{}_{a}{}_{x}-y{}_{m}{}_{i}{}_{n}$3:$div_{x}\leftarrow width/constant_{w}{}_{i}{}_{d}{}_{t}{}_{h}$4:$div_{y}\leftarrow height/constant_{h}{}_{e}{}_{i}{}_{g}{}_{h}{}_{t}$5:**for each **(x,y)∈(X,Y)**do**6:    $x:=x/div_{x}$7:    $y:=y/div_{y}$8:**end for**9:**return** new values for X and Y

As shown in Figure 6A, the bodies are at different distances. By extracting their points from MediaPipe as in Figure 6B, it is clear that the sizes of the bodies are not unified. After doing the depth processing, all bodies are now unified in size and distance (depth) as shown in Figure 6C, and the model can recognize signs accurately.

#### 3.4.2. Shift (Relocation Processing)

**Figure 7:**
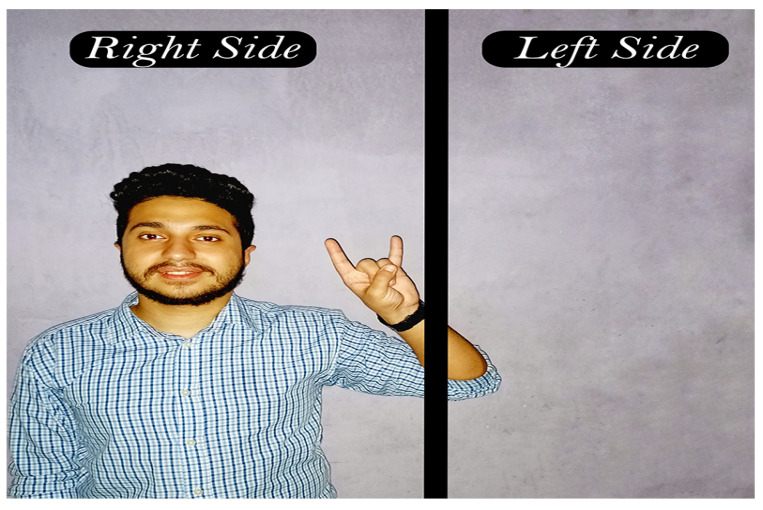
Body on the right-side of the frame.

**Figure 8:**
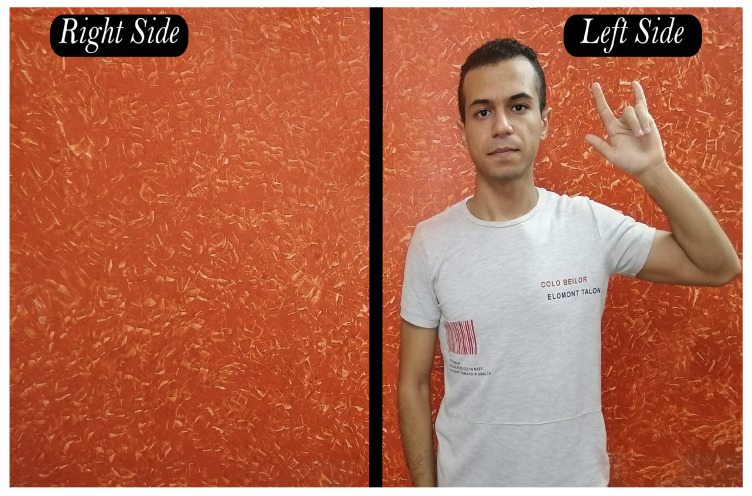
Body on the left-side of the frame.

**Figure 9:**
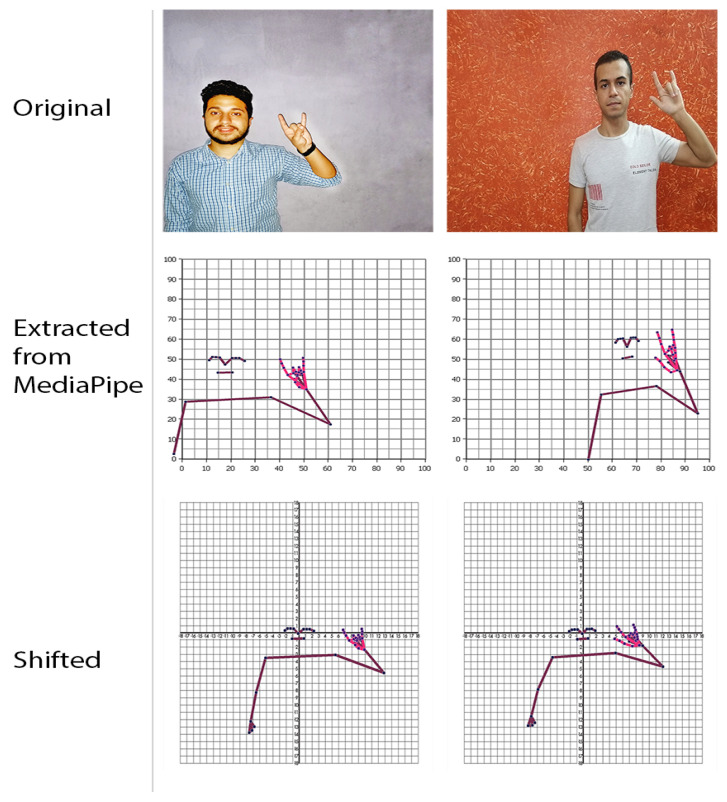
Relocation processing.

In this step, a new challenge appears which is the location of the body in the dataset videos. Suppose a scenario in which the model was trained on a sign in Figure 7 where the body always appears on the far-right side of the frame, and the model was tested on a sign in Figure 8, where the body always appears on the far-left side of the frame. It is obvious that the model will misidentify the sign because of the disparity between training and testing signs. As demonstrated in Figure 9, the body was shifted to be centered by the nose at the point (0,0) in order to fix the problem.

After the two preprocessing phases were finished, it was obtained that the dataset size has been increased while avoiding weakness at any sign, all dataset videos are balanced and unified, and the issues of body distance (depth) and body positioning have been resolved.

### 3.5. The Models

Currently, all of the data are ready to be used as input for the models. The RNN-related model (GRU) and the CNN-related model (1DCNN) are two deep learning models that are included in the solution that our work suggests. In this section, the GRU and 1DCNN models are described together with information on their layers, structure, and data entry methods.

#### 3.5.1. RNN (GRU) Model

A recurrent neural network (RNN) is a type of neural network that analyzes time series or sequential data [34]. It is distinguished by having a “memory”, which enables it to use data from earlier inputs to influence the present input and output [35].

The gated recurrent unit (GRU) is a form of RNN [36]. A forget gate on the GRU makes it resemble an LSTM. LSTM is more accurate when working with datasets that contain longer sequences, but GRU is faster and uses fewer memory [37]. Additionally, GRUs address the problem of vanishing gradients that affects conventional recurrent neural networks [38].

**Figure 10:**
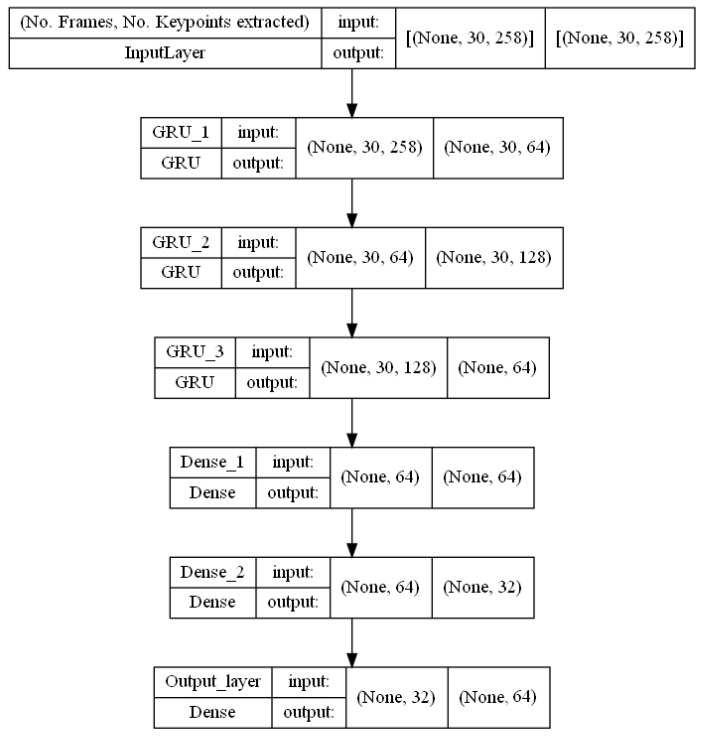
GRU Model structure.

Since the number of frames taken from each video in our case is just 30, it was preferable to utilize the GRU. Table 1 shows the order of the GRU layers with the values that were used in the experiments. The structure of these layers stacked on top of one another is depicted in Figure 10.

#### 3.5.2. 1DCNN Model

Convolutional neural networks (CNNs) are a particular class of neural networks that have excelled in several computer vision tasks and are becoming more and more common in various fields. Convolution layers, pooling layers, and fully connected layers are some of the essential parts of CNNs, which are designed to automatically and flexibly learn and give advanced data using backpropagation [39,40].

**Figure 11:**
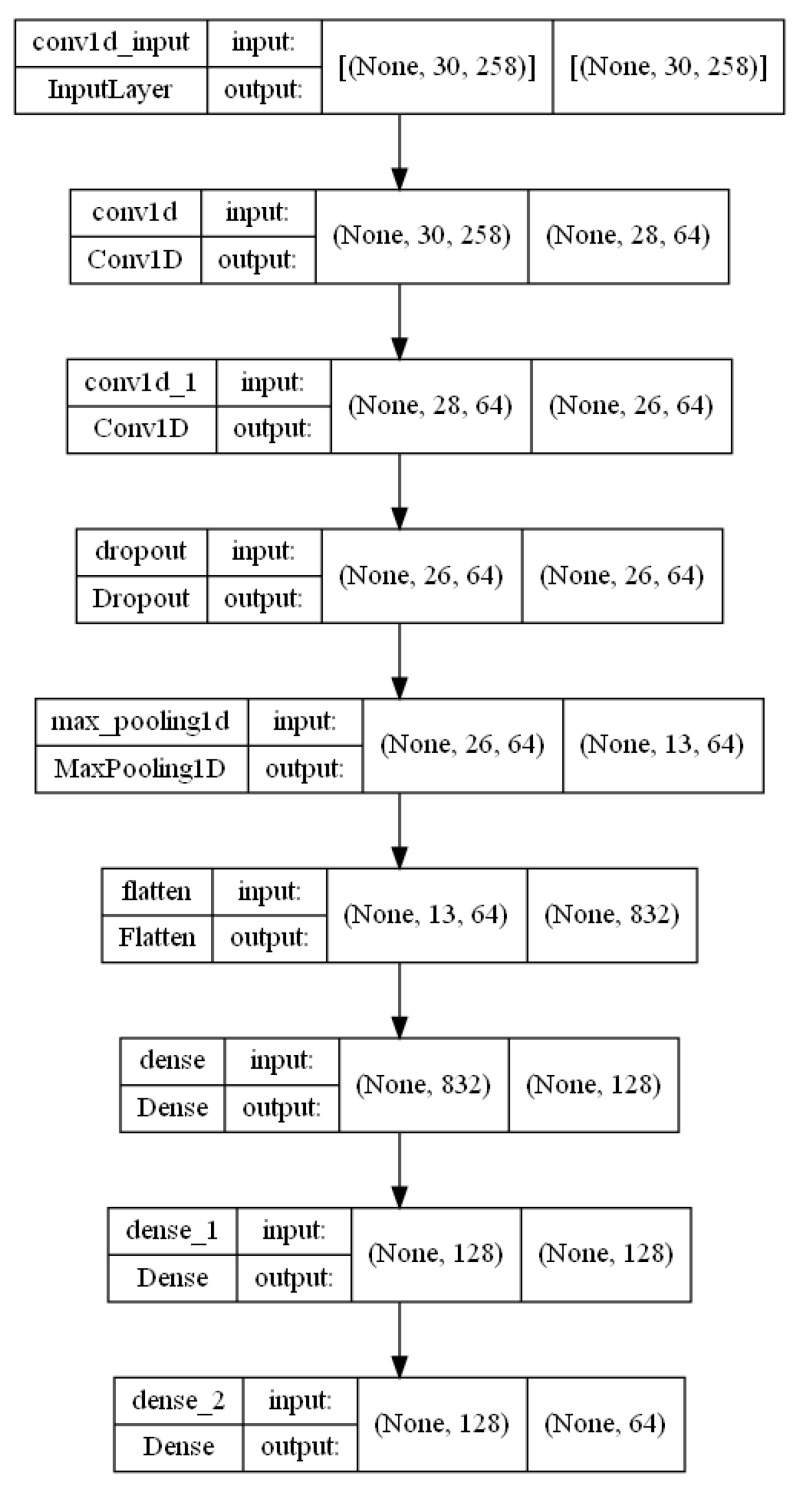
1D CNN model structure.

CNN is a great choice for dynamic sign recognition because the input sample is a 2D matrix, the rows denote the number of frames, and the columns denote the extracted keypoints [41]. The experiments were carried out on a 1D CNN, but they can also be carried out with a 2D CNN, where the 2D CNN is represented as a matrix and the 1D CNN as a vector. Table 2 represents the layers and hyperparameters used in the construction of the 1D CNN model. Figure 11 represents the complete structure used in the experiments for the 1D CNN model.

The models’ architectures were selected in our work after we implemented numerous experiments in which we modified the architectures of the models by changing their number of layers, parameters, optimizers, and activation functions. This is a collection of the values that have been tried, as shown in Table 3, as an example.

## 4. DSL-46 Dataset

The authors of this study generated a dataset (DSL-46) for American sign language that contains 46 frequently used signs that may be used in challenges involving dynamic sign language recognition. None of the participants who contributed to this dataset are highly skilled signers or sign language users. Participants were taught to watch expert records and mimic them before starting to make this dataset.

The DSL-46 dataset contains 2910 videos in total that were recorded in two sets. In the first set, 10 signs were recorded, while, in the second, 36 more signs were added. In Table 4, the signs of the first set start from the sign ‘Hello’ at ID (1) and end with the sign ‘Wear’ at ID (10), while the signs of the second set start from the sign ‘Again’ at ID (11) and end with the sign ‘Wrong’ at ID (46).

The first set was recorded by five participants in an indoor environment with normal light, each participant recorded 15 videos for each sign which led to 750 videos in total. The second set was recorded by four participants in another indoor environment with another source of light to differentiate between the signs and increase the validity of the model. Each participant in the second set recorded 15 videos for each sign also, leading to 2160 videos in total.

By taking some videos with their right hand and others with their left, or even both hands, the participants in both sets attempted to maximize the variety of signs. Additionally, the participants recorded the signs while standing or sitting and against various backgrounds.

An Oppo Reno 3 Pro mobile camera was used in recording all the videos. The duration of DSL-46 videos is 1 second, recorded in 640 by 480 resolution at 30 fps.

## 5. Experiments

### 5.1. Types of Datasets

#### 5.1.1. DSL-46

We already discussed the contents of that dataset and all of its information in Section 4, and now we will go through how to split it in order to enter it into experiments. These data are made up of 2910 videos that were randomly allocated as 60% train, 20% validation, and 20% test, giving us 1746 videos for train, 582 videos for validation, and 582 videos for test.

#### 5.1.2. LSA64

Many experiments were conducted on the LSA64 dataset to evaluate the effectiveness of our models. LSA64 is a database for the Argentinian sign language; it produces a dictionary that includes 3200 videos; ten subjects executed five repetitions of 64 different types of signs. Signs were selected among the most used ones in the LSA lexicon, including both verbs and nouns [14].

Each sign was executed by imposing a few constraints on the subjects to increase diversity and realism in the database. All subjects were non-signers and right-handed and were taught how to perform the signs during the recording session by showing them a video of the signs as performed by one of the authors and practicing each sign a few times before recording.

To conduct the experiments, the authors performed a subject-dependent classification by splitting the dataset randomly into 1920 videos for training, 640 videos for validation, and 640 videos for testing. Each set is created randomly to avoid bias factors.

#### 5.1.3. LIBRAS-BSL

The LIBRAS-BSL dataset, which is a collection of 37 signs from the Brazilian Sign Language, is used to train deep learning models for dynamic sign language recognition tasks. A total of 10 participants—6 females and 4 males—created 4440 videos. Each participant performed 12 times of 37 different signs. In the LIBRAS-BSL dataset, RGB, Depth, and skeleton data were gathered using a Microsoft Kinect [8,15].

### 5.2. Experiments Environment

The experiments were carried out on two distinct machines, one of which used the CPU and the other the GPU. The experiments were carried out using the CPU on the first device, an Intel Core i3-10100 PC with a 3.6 GHz clock speed, 16 GB of RAM, and a Crucial P5 Plus 500 GB SSD. The experiments were carried out using the graphics card (GPU) on the second device, a laptop with a 2.5 GHz Intel Core i7-7700HQ CPU, 6 GB of NVIDIA GTX 1060 GPU, 16 GB of RAM, and a 256 GB SSD.

### 5.3. Experiments’ Results

Five experiments were carried out to calculate the mean accuracy using three different datasets (Section 5.1). We randomly separated the dataset into three subsets: training, validation, and testing (60 percent training, 20 percent validation, and 20 percent testing).

In the following tables ( Table 5, Table 6 and Table 7), the mean accuracy for training, validation, and testing will be clarified for each used model, and whether it includes the pose points or not.

Table 5 shows those experiments on our DSL-46 dataset while Table 6 on the LSA64 dataset and Table 7 on the LIBRAS-BSL dataset.

Determining the position of the hand relative to the body helps in differentiating between signs, as two or more signs can be made with the same hand shape, but the different locations relative to the body change the meaning of the sign. Therefore, in order to determine the location of the hand in relation to the body, use one of the two ways. The first way is the pose keypoints, which are used to determine where the hand is in relation to the body. The second way is by excluding the pose key points, but, before that, we move the body to the point (0,0) on the *x*- and *y*-axes (see Section 3.4.2). Thus, the position of the hand will be known in relation to the axes.

Therefore, the authors considered conducting an experiment to see if the pose points might be eliminated following this preprocessing. Since the relocation is processed in Section 3.4.2 using the pose points, it should not be dispensed before this stage.

### 5.4. Discussion

We initially draw the conclusion that the proposed system work is effective based on prior experiments. Second, CNN is more effective than the GRU, and the inclusion points of the pose are sometimes given with a 1% higher accuracy and, other times, there is no difference, as we see in experiment No. 4 that does not contain pose points, so we find that, in the test accuracy, it is higher than Experiment No. 1 and equal to No. 2, and they are the ones who include the pose points. The comparison between these three tables (Table 5, Table 6 and Table 7 ) shows that the significant degree of similarity displayed in the primary parameters of the LIBRAS-BSL dataset is one of its difficulties.

### 5.5. Comparison with State-of-the-Art SLR Methods

The mean accuracy calculated from five experiments using the LSA64 and LIBRAS-BSL datasets, respectively, is summarized in Table 8 and Table 9.

Here, we will compare a series of research studies that used the LSA64 and LIBRAS-BSL datasets to recognize sign languages, and we will compare the numbers of our findings to theirs. A comparison with state-of-the-art SLR methods has been executed under the same conditions on the datasets, with the same training and test partitions. This comparison shows that, despite the simplicity of our method, it outperforms competitors in accuracy, although they employ more complicated and demanding networks.

In Table 8, it is clear that our results, especially in the 1D-CNN, are more accurate than the other techniques and are equal to No. 6 [33] and that the No. 9 [8] technique gives a greater result than us, but let us talk about the differences that distinguish us from No. 6 [33] and No. 9 [8], so we can almost be certain and not confirm that our technology is much faster and lighter the others, which they use complex techniques to identify.

Let us start with No. 6 [33] first, as they use a mixture of VGG16, FlowNet, and OpenPose, and it has been proven in Valentin et al. [13] that the MediaPipe is faster than the OpenPose with an average between 25 and 75 times. In addition, the VGG16 structure is more complex and requires more layers than 1D-CNN [42]. Training VGG-16 on the DSL-46 dataset takes 1 h 12 m 15 s, while the training time of 1D-CN on the same dataset is 39 m 40 s. In the second paper [8], in this research, they use three methods, first 3S-CNN and second 2S-CNN-C while third Later_Fusion (3S + 2SC). In the first and second method, although they use more complex models and methods than us, our results were higher, while the third method, which is higher than ours, relies on very complex processes to recognize it. They use a combination of two prediction models, the first consists of three stream CNN and the second consists of two stream CNN, while we only use one stream CNN, which makes us see the power difference between the complexities of the models. Besides all that, all the research in the table is concerned with identifying sign language in its ideal form only in the dataset. However, our paper is more concerned with the accuracy of the recognition during real-time because in real-time the ideal environment is not available.

### 5.6. Limitations

Despite the fact that our method has very high accuracy in recognizing signs and a preprocessing algorithm to deal with the scale of the bodies, the location, and the duration of the input videos, as with any work, there must be limitations. The limitation of our research is that the dataset size is still not big enough, the model faces difficulties in separating signs with the movement speed of hands in real-time, and the anomaly signs could be detected as signs, and this is wrong.

## 6. Conclusions

In this paper, we propose lightweight deep learning models combined with the MediaPipe framework based on pre-processing techniques, e.g., location and depth processing, to increase the performance of our approach, especially in real-time. Our approach can locate the hands by determining their shape, direction, and position. relative to the body in each frame. Then, we use these data as input for the GRU model from the RNN class and the CNN model to train from the landmarks features to be able to recognize signs even in real-time streams.

Our experiments were conducted on three different sign language datasets. The first one is DSL-46, which is our dataset for the American sign language, the second one is LSA64 for Argentinian sign language, while the third is the LIBRAS-BSL dataset for Brazilian sign language.

The result of the experiments showed that our solution method succeeded in recognizing dynamic signs with an accuracy of more than 99% on both datasets. Comparisons were made with other papers that used the same dataset, and the results were discussed. The include and exclude of the pose were tested by showing the difference between them.

The main conclusions of this paper can be summarized as follows: deep learning models combined with the MediaPipe framework were used to create a lightweight model for dynamic sign language recognition, and a pre-processing technique was developed to adapt the model for recognizing signs in a real-world video. A new dataset of dynamic sign language named DSL-46 with 46 vocabularies was recorded, and 12 experiments on the GRU model and the 1D CNN model were conducted. The results show that 1D CNN performs better than GRU in both training and testing, and it was found that, in most of the author’s experiments, including the pose keypoints increases the accuracy by 1%.

In the future, we will work on recording more signs and expanding the dataset size, working on the difficulties in separating signs with the movement speed of hands in real-time detection, creating a lighter model to be compatible with mobile devices, comparing the DSL46 with state-of-the-art methods, and make a complex real-time sign sentence detector application.

## Figures and Tables

**Table 1:** GRU model layers and parameters.

Layer	Type	Number of Neurons	Activation
In	Input	(n_frame, n_keypoints)	-
$G_{1}$	GRU	64	ReLU
$G_{2}$	GRU	128	ReLU
$G_{f}$	GRU	64	ReLU
$D_{1}$	Dense	64	ReLU
$D_{2}$	Dense	32	ReLU
Out	Output	Number of classes	Softmax

**Table 2:** 1D CNN model layers & parameters.

Layer	Type	Size	Kernel Size	Stride	Padding	Activation
In	Input	(n_frame, n_keypoints)	-	-	-	-
$C_{1}$	Convolution	64	3	1	Valid	ReLU
$C_{2}$	Convolution	64	3	1	Valid	ReLU
$D_{1}$	Droput	0.9	-	-	-	-
$M_{1}$	MaxPooling1D	2	-	2	valid	-
$F_{1}$	Fully Connected	128	-	-	-	ReLU
$F_{2}$	Fully Connected	128	-	-	-	ReLU
Out	Output	Number of classes	-	-	-	Softmax

**Table 3:** Collection of values that have been tried with model layers.

Layer	Value
GRU or CNN layers	Between 1 to 3
Dropout layers	Between 1 to 3
Dropout layer value	Between 0.5 to 0.9
Neurons per layer	Between 32 to 256
Hidden layers	Between 1 to 5
Activation	ReLU or leakyReLU
Output Activation	Softmax
Optimizer	Adam or Adamax

**Table 4:** DSL-46 dataset SIGNS.

ID	Name	ID	Name
1	Hello	24	Name
2	How Are	25	Not
3	Love	26	Right
4	No	27	Come
5	Please	28	Father
6	Mask	29	Give to
7	You	30	Hearing
8	Sorry	31	Learn
9	Thanks	32	Me
10	Wear	33	Need
11	Again	34	Ours
12	Don’t Want	35	Sad
13	Finish	36	Work
14	Good	37	Deaf
15	Help	38	Fine
16	More	39	Go
17	Sign	40	Like
18	We	41	Meet
19	Yes	42	Nice
20	Age	43	Pay
21	Eat	44	See you later
22	Forget	45	Want
23	Happy	46	Wrong

**Table 5:** Experiments results on the DSL-46 dataset.

Exp No.	Includes Pose Keypoints	Model Type	Train Accuracy	Validation Accuracy	Test Accuracy
1	Yes	GRU	100	96.74	97.08
2	Yes	CNN	100	98.78	98.80
3	No	GRU	99.42	95.2	96.91
4	No	CNN	99.88	99.1	98.80

**Table 6:** Experiments’ results on the LSA64 dataset.

Exp No.	Includes Pose Keypoints	Model Type	Train Accuracy	Validation Accuracy	Test Accuracy
5	Yes	GRU	99.89	97.65	97.96
6	Yes	CNN	100	99.21	99.84
7	No	GRU	99.79	97.18	96.87
8	No	CNN	99.73	98.90	98.75

**Table 7:** Experiments results on the LIBRAS-BSL dataset.

Exp No.	Includes Pose Keypoints	Model Type	Train Accuracy	Validation Accuracy	Test Accuracy
9	Yes	GRU	89.58	87.75	87.86
10	Yes	CNN	90.12	89.29	88.40
11	No	GRU	87.73	87.18	86.87
12	No	CNN	88.23	87.10	86.95

**Table 8:** Comparison with state-of-the-art SLR methods on the LSA64 Dataset.

No.	Method	Mean Accuracy
1	3DCNN [30]	93.90
2	ALL-BF-SVM [29]	95.08
3	ALL-HMM [29]	95.92
4	ALL(sequence agnostic) [29]	97.44
5	Deep network [32]	98.09
6	VGG16+FlowNet+OpenPose [33]	99.84
7	3S-CNN [8]	96.92
8	2S-CNN-C [8]	99.82
9	Later Fusion (3S + 2SC) [8]	99.91
10	**GRU**	**97.96**
11	**1D-CNN**	**99.84**

**Table 9:** Comparison with state-of-the-art SLR methods on the LIBRAS-BSL dataset.

No.	Method	Mean Accuracy
12	3S-CNN [8]	79.95
13	2S-CNN-C [8]	81.12
14	Later Fusion (3S + 2SC) [8]	84.71
15	**GRU**	**87.86**
16	**1D-CNN**	**88.40**

**Figure A1:**
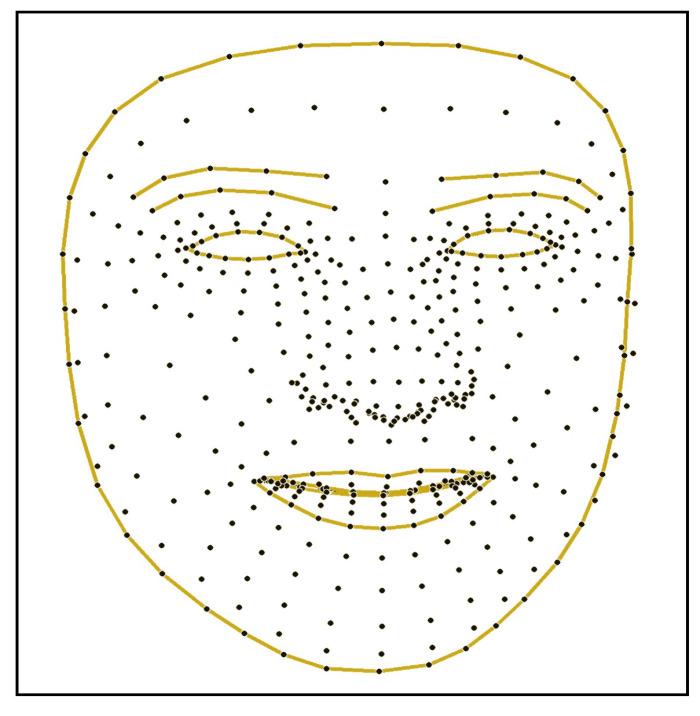
Face Mesh keypoints [20].

## Data Availability

The DSL46-dataset presented in this study is openly available and can be accessed in OSF at: https://osf.io/t92sd (accessed on 10 November 2022).

## Appendix A

This section demonstrates the face keypoints inclusion approach using the Face mesh method from MediaPipe. The MediaPipe Face Mesh is an approach to extract 3D landmarks from the human face, with 468 keypoints for each dimension with a total of 1404 keypoints as shown in Figure A1. Real-time identification is also possible with the Face Mesh technology, and it can also distinguish between distinct facial expressions that aid in accurately recognizing sign language. The Face Mesh approach has been tested in the past in our previous work [20], where we conducted six experiments to compare the effects of utilizing and not using the Face Mesh in DSLR. In conclusion, the research findings indicate that it is possible to accurately recognize signs by determining the facial expression using the Face Mesh approach. However, a speedier recognizer model was created as a result of the Face Mesh method’s abandonment since it saves 468 keypoints in 3D (1404 keypoints).

## References

[1] https://www.who.int/news-room/fact-sheets/detail/deafness-and-hearing-loss.

[2] Alaghband M., Yousefi N., Garibay I. (2021). Facial Expression Phoenix FePh An Annotated Sequenced Dataset for Facial and Emotion Specified Expressions in Sign Language. Eng. World.

[3] Theodorakis S., Pitsikalis V., Maragos P. (2014). Dynamic–Static unsupervised sequentiality, statistical subunits and lexicon for sign language recognition. Image Vis. Comput..

[4] Abdalla M.S., Hemayed E.E. (2013). Dynamic hand gesture recognition of arabic sign language using hand motion trajectory features. Glob. J. Comput. Sci. Technol..

[5] Cheok M.J., Omar Z., Jaward M.H. (2019). A review of hand gesture and sign language recognition techniques. Int. J. Mach. Learn. Cybern..

[6] Wadhawan A., Kumar P. (2012). Sign Language Recognition Systems: A Decade Systematic Literature Review. Arch. Comput. Methods Eng..

[7] Rastgoo R., Kiani K., Escalera S. (2021). Sign language recognition: A deep survey. Expert Syst. Appl..

[8] Escobedo E., Ramirez L., Camara G. Dynamic Sign Language Recognition Based on Convolutional Neural Networks and Texture Maps. Proceedings of the 2019 32nd SIBGRAPI Conference on Graphics, Patterns and Images (SIBGRAPI).

[9] Liao Y., Xiong P., Min W., Min W., Lu J. (2019). Dynamic sign language recognition based on video sequence with blstm-3d residual networks. IEEE Access.

[10] Chaikaew A., Somkuan K., Yuyen T. Thai sign language recognition: An application of deep neural network. Proceedings of the 2021 Joint International Conference on Digital Arts, Media and Technology with ECTI Northern Section Conference on Electrical, Electronics, Computer and Telecommunication Engineering.

[11] Hoang M.T., Yuen B., Dong X., Lu T., Westendorp R., Reddy K. (2019). Recurrent Neural Networks for Accurate RSSI Indoor Localization. IEEE Int. Things J..

[12] Zhang F., Bazarevsky V., Vakunov A., Tkachenka A., Sung G., Chang Ch., Grundmann M. (2020). MediaPipe Hands: On-device Real-time Hand Tracking. arXiv.

[13] Bazarevsky V., Grishchenko I., Raveendran K., Zhu T., Zhang F., Grundmann M. (2020). BlazePose: On-device Real-time Body Pose tracking. arXiv.

[14] De Giusti L.C., Chichizola F., Rodriguez Eguren S., Sánchez M., Paniego J.M., De Giusti A.E. LSA64: An Argentinian sign language dataset. Proceedings of the XXII Congreso Argentino de Ciencias de la Computación (CACIC 2016).

[15] Cerna L.R., Cardenas E.E., Miranda D.G., Menotti D., Camara-Chavez G. (2020). A multimodal LIBRAS-UFOP Brazilian sign language dataset of minimal pairs using a microsoft Kinect sensor. Expert Syst. Appl..

[16] Sonawane T., Lavhate R., Pandav P., Rathod D. (2017). Sign language recognition using leap motion controller. Int. J. Adv. Res. Innov. Ideas Edu..

[17] Li K., Zhou Z., Lee C.H. (2016). Sign transition modeling and a scalable solution to continuous sign language recognition for real-world applications. ACM Trans. Access. Comput. (TACCESS).

[18] Yang X., Chen X., Cao X., Wei S., Zhang X. (2016). Chinese sign language recognition based on an optimized tree-structure framework. IEEE J. Biomed. Health Inform..

[19] Liu T., Zhou W., Li H. Sign language recognition with long short-term memory. Proceedings of the 2016 IEEE International Conference on Image Processing (ICIP).

[20] Samaan G.H., Wadie A.R., Attia A.K., Asaad A.M., Kamel A.E., Slim S.O., Abdallah M.S., Cho Y.-I. (2022). MediaPipe’s Landmarks with RNN for Dynamic Sign Language Recognition. Electronics.

[21] Cardenas E.E., Camara-Chavez G. (2017). Fusion of Deep Learning Descriptors for Gesture Recognition Iberoamerican Congress on Pattern Recognition.

[22] Pigou L., Dieleman S., Kindermans P.-J., Schrauwen B. (2014). Sign Language Recognition Using Convolutional Neural Networks Workshop at the European Conference on Computer Vision.

[23] Camgoz N.C., Hadfield S., Koller O., Bowden R. Using convolutional 3d neural networks for user-independent continuous gesture recognition. Proceedings of the 2016 23rd International Conference on Pattern Recognition (ICPR).

[24] ElBadawy M., Elons A., Shedeed H.A., Tolba M. Arabic sign language recognition with 3D convolutional neural networks Intelligent computing and information systems (ICICIS). Proceedings of the 2017 Eighth International Conference on Tools with Artificial Intelligence, IEEE.

[25] Pu J., Zhou W., Li H. Dilated convolutional network with iterative optimization for continuous sign language recognition. Proceedings of the Twenty-Seventh International Joint Conference on Artificial Intelligence (IJCAI-18).

[26] Rao G.A., Syamala K., Kishore P., Sastry A. Deep convolutional neural networks for sign language recognition. Proceedings of the 2018 Conference on Signal Processing in Addition, Communication Engineering Systems (SPACES).

[27] Cui R., Liu H., Zhang C. Recurrent convolutional neural networks for continuous sign language recognition by staged optimization. Proceedings of the IEEE Conference on Computer Vision and Pattern Recognition.

[28] Gupta P.M.X.Y.S., Kautz K.K.S.T.J. Online Detection and Classification of Dynamic Hand Gestures with Recurrent 3d Convolutional Neural Networks. Proceedings of the IEEE Conference on Computer Vision and Pattern Recognition.

[29] Ronchetti F., Quiroga F., Estrebou C., Lanzarini L., Rosete A., Montes y Gómez M., Escalante H., Segura A., Murillo J. (2016). Sign Languague Recognition Without Frame-Sequencing Constraints: A Proof of Concept on the Argentinian Sign Language. Advances in Artificial Intelligence—IBERAMIA 2016.

[30] Neto G.M.R., Junior G.B., de Almeida J.D.S., de Paiva A.C., Campilho A., Karray F., ter Haar Romeny B. (2018). Sign Language Recognition Based on 3D Convolutional Neural Networks. Image Analysis and Recognition. ICIAR 2018.

[31] Molchanov P., Gupta S., Kim K., Pulli K. Multi-sensor system for driver’s hand-gesture recognition. Proceedings of the 2015 11th IEEE International Conference and Workshops on Automatic Face and Gesture Recognition (FG).

[32] Konstantinidis D., Dimitropoulos K., Daras P. Sign Language Recognition Based on Hand and Body Skeletal Data. Proceedings of the 2018-3DTV-Conference: The True Vision—Capture, Transmission and Display of 3D Video (3DTV-CON).

[33] Konstantinidis D., Dimitropoulos K., Daras P. A Deep Learning Approach for Analyzing Video and Skeletal Features in Sign Language Recognition. Proceedings of the 2018 IEEE International Conference on Imaging Systems and Techniques (IST).

[34] Zhang Z., Wu G., Li Y., Yue Y., Zhou X. Deep Incremental RNN for Learning Sequential Data: A Lyapunov Stable Dynamical System. Proceedings of the 2021 IEEE International Conference on Data Mining (ICDM).

[35] Sherstinsky A. (2018). Fundamentals of Recurrent Neural Network (RNN) and Long Short-Term Memory (LSTM) Network. Phys. Nonlinear Phenom..

[36] Chung J., Gulcehre C., Cho K., Bengio Y. (2014). Empirical Evaluation of Gated Recurrent Neural Networks on Sequence Modeling. arXiv.

[37] Cahuantzi R., Chen X., Güttel S. (2021). A comparison of LSTM and GRU networks for learning symbolic sequences. arXiv.

[38] Mateus B.C., Mendes M., Farinha J.T., Assis R., Cardoso A.M. (2021). Comparing LSTM and GRU Models to Predict the Condition of a Pulp Paper Press. Energies.

[39] O’Shea K., Nash R. (2015). An Introduction to Convolutional Neural Networks. arXiv.

[40] Albawi S., Mohammed T.A., Al-Zawi S. Understanding of a convolutional neural network. Proceedings of the 2017 International Conference on Engineering and Technology (ICET).

[41] Mostavi M., Chiu Y.C., Huang Y., Chen Y. (2020). Convolutional neural network models for cancer type prediction based on gene expression. BMC Med. Genom..

[42] Alzubaidi L., Zhang J., Humaidi A.J., Al-Dujaili A., Duan Y., Al-Shamma O., Santamaría J., Fadhel M.A., Farhan L. (2021). Review of deep learning: Concepts, CNN architectures, challenges, applications, future directions. J. Big Data.

